# The relationship between blood lead levels and occupational exposure in a pregnant population

**DOI:** 10.1186/s12889-016-3902-3

**Published:** 2016-12-07

**Authors:** Osmel La-Llave-León, José Manuel Salas Pacheco, Sergio Estrada Martínez, Eloísa Esquivel Rodríguez, Francisco X. Castellanos Juárez, Ada Sandoval Carrillo, Angélica María Lechuga Quiñones, Fernando Vázquez Alanís, Gonzalo García Vargas, Edna Madai Méndez Hernández, Jaime Duarte Sustaita

**Affiliations:** 1 Instituto de Investigación Científica, Universidad Juárez del Estado de Durango, Avenida Universidad esq. con Volantín, Zona Centro, C.P. 34000 Durango, DGO Mexico; 2 Facultad de Enfermería y Obstetricia, Universidad Juárez del Estado de Durango, Ave. Cuauhtémoc, 223 norte, CP 34 000 Durango, Mexico; 3 Hospital General de Durango, Secretaría de Salud de Durango, Durango, Mexico; 4 Facultad de Medicina de Gómez Palacio, Universidad Juárez del Estado de Durango, La Salle 1 y Sixto Ugalde, S/N. Col. Revolución, CP. 35050 Gómez Palacio Durango, Mexico

**Keywords:** Blood lead, Occupational exposure, Pregnant women, Risk factors

## Abstract

**Background:**

Pregnant women exposed to lead are at risk of suffering reproductive damages, such as miscarriage, preeclampsia, premature delivery and low birth weight. Despite that the workplace offers the greatest potential for lead exposure, there is relatively little information about occupational exposure to lead during pregnancy. This study aims to assess the association between blood lead levels and occupational exposure in pregnant women from Durango, Mexico.

**Methods:**

A cross-sectional study was carried out in a population of 299 pregnant women. Blood lead was measured in 31 women who worked in jobs where lead is used (exposed group) and 268 who did not work in those places (control group). Chi-square test was applied to compare exposed and control groups with regard to blood lead levels. Odds ratio (OR) and 95% confidence intervals (CI) were calculated. Multivariable regression analysis was applied to determine significant predictors of blood lead concentrations in the exposed group.

**Results:**

Exposed women had higher blood lead levels than those in the control group (4.00 ± 4.08 μg/dL vs 2.65 ± 1.75 μg/dL, *p* = 0.002). Furthermore, women in the exposed group had 3.82 times higher probability of having blood lead levels ≥ 5 μg/dL than those in the control group. Wearing of special workwear, changing clothes after work, living near a painting store, printing office, junkyard or rubbish dump, and washing the workwear together with other clothes resulted as significant predictors of elevated blood lead levels in the exposed group.

**Conclusions:**

Pregnant working women may be at risk of lead poisoning because of occupational and environmental exposure. The risk increases if they do not improve the use of protective equipment and their personal hygiene.

**Electronic supplementary material:**

The online version of this article (doi:10.1186/s12889-016-3902-3) contains supplementary material, which is available to authorized users.

## Background

Lead has been clearly shown to be a neurotoxic agent widely distributed in the environment [1]. Excessive lead exposure may occur in the workplace. Some jobs that expose people to lead include: mining, smelting, foundry work, construction, plumbing, radiator manufacturing, lead-acid battery recycling, manufacturing of rubber products, and the chemical industry. Years ago, lead was also used regularly in paint, ceramics, and pipe solder among other things. Because of its potential health problems, the amount of lead used in these products today has lessened or has been removed. However, lead is still common in many industries, including construction, mining, and manufacturing [2].

Lead can harm many of the body’s organ systems. Human exposure to lead can result in a wide range of biological effects [3]. It is well known that childhood and pregnancy are the most sensitive population to lead exposure. A pregnant woman with an elevated blood lead concentration may expose her fetus to the toxic effect of lead. Elevated blood lead levels (BLLs) in children cause learning and behavioral deficits [4, 5]. Low-level lead exposure, including prenatal exposure, has been linked to decreased performance on IQ tests for school children [6–9]. Several studies have suggested that any level of exposure is potentially detrimental and no threshold for these effects has been identified [10, 11].

Lead concentrations have declined in the last decades due to the increase in health interventions [12]. In spite of this, lead exposure remains a risk factor for female reproductive health, even at low levels of lead in blood [13]. Once absorbed from the gastrointestinal tract or the respiratory system, lead is transported bound to erythrocytes and accumulates in bone [14]. During pregnancy, calcium demands increase. This leads to increased bone turnover, with a consequential release of lead from bone and increased blood lead levels [15, 16]. Lead can cross the placenta and expose the fetus to the harmful effects of this toxic, thus affecting the embryonic development of multiple organs and causing neurobehavioral impairments in infancy and early childhood [4, 5, 9, 17]. Therefore, pregnancy is considered a critical time for exposure to lead for the mother and the fetus [14, 18].

Over the past several decades there has been a remarkable reduction in environmental sources of lead and a decreasing trend in the prevalence of elevated blood lead levels [2]. However, some reproductive health damages at levels of lead in blood below 10 μg/dL have been reported. Therefore, in recent years, many studies have focused on the health effects at low levels of lead in blood. Low blood lead concentrations in pregnant women have been associated with miscarriage [19, 20], pregnancy hypertension, or preeclampsia [12, 21–24] premature delivery [13], premature rupture of the membranes [25], and low birth weight [26, 27]. On the other hand, it is considered that lead-related toxicity can occur at levels as low as 5 μg/dL [28]. Hence, maternal exposure to lead plays an important role in adverse pregnancy outcomes.

Despite that the workplace offers the greatest potential for lead exposure, there is relatively little information about the occupational exposure to lead during pregnancy. It is necessary to identify sources of lead exposure relevant to this population. Some of the jobs that commonly involve lead exposure are battery manufacture or repair; construction (welding or cutting lead-painted metal); radiator manufacture or repair; wire cable cutting and manufacture, and cable, battery, or scrap metal salvage, plating operations; manufacturing or using leaded paints, dyes or pigments, or lead soldering in the electronics industry, among others [29]. In Mexico, and in other developing countries, it is common to find pregnant women working in places with potential sources of lead exposure. The aim of this study was to assess the association between blood lead levels and occupational exposure in pregnant women from Durango, Mexico.

## Methods

### Study population

From June 2007 to May 2008 a cross-sectional study was conducted to evaluate the association between BLLs and some risk factors in pregnant women who received health attention in the State of Durango, Mexico [30]. The study population consisted of pregnant women who received medical attention in two sanitary jurisdictions pertaining to the Secretary of Health. The total estimated number of pregnant women seen in these two jurisdictions during a 1 year period was obtained from the Secretariat of Health databases, and the sample required was distributed equally in 12 municipalities. The participants were recruited from Obstetrics and Gynecology Departments of the municipal hospitals. All women who presented for prenatal care on the days that the study team visited, independent of their gestational age, were asked to participate in the study if they met the inclusion criteria. The inclusion criteria were: being pregnant, living in Durango, able to understand Spanish, and receiving health care paid for by the Secretary of Health. Each municipality was visited two or three times during the recruitment period, until the sample size was completed. Of the 337 pregnant women who presented for prenatal care on the days of the visits, 12 women were excluded because they did not live in Durango and 26 declined to participate in the study. A total of 299 women were included in the study (Aditional file 1). The interviewer’s interaction with patients was standardized. All patients gave their informed written consent and answered a set of questions in a face-to-face interview. The research protocol was approved by the Ethical Committee of Durango General Hospital.

First, the group was treated as a cohort. After that, a regression with lead levels as outcome allowed to attribute the proportion of risk from occupational and non-occupational exposure. For assessment of the association between blood lead levels and occupational exposure, subjects were classified into two groups: women who worked in places where lead is used (exposed group) and women who did not work in those places (control group). Women who worked in automotive repair shops, mining laboratories, welding workshops, automotive harness factories, hairdressing salons, and road sweepers were included in the exposed group. Unemployed women and those women who had a job where lead-containing materials are not used, were included in the control group.

### Blood lead measurement

Blood samples were collected using lead-free tubes containing EDTA. Samples were stored in the original tube at 4^o^ C before being transferred to the Environmental Toxicology Laboratory, Faculty of Medicine, Juarez University of Durango State. The time between receipt and analysis varied from 1 to 3 weeks. During which time, the specimens were stored refrigerated at 4 °C. Lead concentration was determined by graphite furnace atomic absorption spectrometry. Bovine blood obtained from the National Institute of Standards and Technology (NIST) was used as standard reference material.

### Statistical analysis

Data were analyzed to describe demographic characteristics, BLLs, and potential sources of lead exposure. The normality of the variables was tested using the Kolmogorov-Smirnov test. BLLs were log-transformed prior to analysis. Multivariable regression analysis was conducted to determine the proportion of risk from each occupational and non-occupational exposure. After that, the study population was divided into two groups according to occupation (occupationally exposed and non-occupationally exposed). Student *t-*test was applied for comparison of quantitative variables. Chi-square test was applied to compare exposed and control groups regarding blood lead levels (BLLs ≥ 5 μg/dL vs BLLs < 5 μg/dL). Odds ratio (OR) and 95% confidence intervals were calculated. To identify non-occupational sources of lead exposure for pregnant women we explored the following: the way in which workwear is washed (together with other clothes or alone), use of lead-glazed pottery, use of hair dyes, living near workplaces where lead is used (mining zones, battery workshops, junkyards, rubbish dumps and painting workshops), pica behavior and living with someone who works with lead, in both exposed and control groups. These activities have been documented to be lead-related. Chi-square test was also used to compare both groups regarding non-occupational sources of lead exposure. Student *t*-test was also used to compare blood lead levels according to some protection habits in the exposed group. Use of respiratory protective equipment, habit of wearing gloves, wearing of special workwear, handwashing before eating, changing clothes after work, and use of any protective equipment were analyzed as dichotomous variables. Finally, backward stepwise multivariable regression analysis was applied to determine significant predictors of blood lead concentrations in the exposed group. A set of variables selected on the basis of previous knowledge or because of associations with lead levels in bivariate analyses (at *p* < 0.25) were entered into the model. The full model was followed by stepwise backward elimination to determine whether each variable remained significant after non-significant covariates were excluded. All statistical analyses were performed using SPSS for Windows statistical package version 15.0. A *p*-value < 0.05 was considered statistically significant.

## Results

The mean blood lead concentration in the study population was 2.79 μg/dL (SD 2.14), geometric mean 2.38 μg/dL, 95% CI (2.25 – 2.54). Among the 299 pregnant women enrolled in the study, 31 (10.4%) worked in places where lead is used, and 268 (89.6%) did not work where lead-containing materials are used (Table 1). Results of multiple linear regression on association between blood lead levels and risk factors are shown in Table 2. Living in a mine zone was associated with increased blood lead (*p* = 0.044). However, working in places where lead is used was the main factor associated with blood lead concentration. On the basis of this result, the study population was divided into two groups: exposed and non-exposed.

**Table 1:** General information and blood lead levels of study population (*N* = 299)

Variables	Percent	Mean (SD)
Age (years)		24.32 (6.71)
Gestational age (weeks)		24.07 (8.68)
Pregnancies		2.0 (1.0)
Body mass index (kg/m^2^)		27.23 (5.63)
Hemoglobin (g/dL)		12.55 (1.34)
Monthly income per person, USD		140.95 (144.73)
Working in places where lead is used		
Yes	10.4	
No	89.6	
Blood lead levels (μg/dL)		2.79 (2.14)
Geometric mean (95% CI)		2.38 (2.25 – 2.54)

**Table 2:** Results from the multiple linear regression analysis on the association between blood lead and risk factors

Risk factor	Coefficient β	95% CI	*p*
Washing the workwear together with other clothes	0.106	- 0.018 – 0.229	0.093
Use of lead glazed pottery	0.033	- 0.102 – 0.168	0.634
Dyeing hair	- 0.016	- 0.147 – 0.115	0.813
Living near workplaces where lead is used	- 0.021	- 0.197 – 0.156	0.818
Living near mining zone	0.237	0.006 – 0.468	0.044
Living near battery workshop	- 0.016	- 0.209 – 0.177	0.869
Living near junkyard	- 0.079	- 0.284 – 0.127	0.452
Living near rubbish dump	0.141	- 0.060 – 0.342	0.169
Living near straightening and painting workshop	0.023	- 0.172 – 0.218	0.819
Pica behavior	0.115	- 0.032 – 0.261	0.124
Living with someone who works with lead	0.056	- 0.071 – 0.183	0.387
Living near painting store	0.081	- 0.167 – 0.329	0.521
Living near printing office	- 0.120	- 0.441 – 0.201	0.461
Working in places where lead is used	0.306	0.103 – 0.509	0.003

R2 = 0.082

Table 3 summarizes the main characteristics of both groups. There were no significant differences between the groups regarding age, gestational age, number of pregnancies, body mass index (BMI), hemoglobin and monthly income per person. However, the blood lead concentration of the exposed group was significantly higher than that of the control group (*p* = 0.002).

**Table 3:** General information and blood lead levels of the exposed subjects and control group^a^

Variable	Exposed group (*n* = 31)	Control group (*n* = 268)	*p* value^*^
Age (years)	26.03 (6.17)	24.13 (6.76)	0.135
Gestational age (weeks)	22.71 (8.06)	24.22 (8.75)	0.358
Number of pregnancies	2.55 (1.38)	2.23 (1.47)	0.253
Body mass index (kg/m^2^)	28.81 (4.79)	27.04 (5.70)	0.098
Hemoglobin (g/dL)	12.97 (1.11)	12.50 (1.36)	0.065
Monthly income per person, USD	165.62 (130.59)	138.00 (146.28)	0.316
Blood lead levels (μg/dL)	4.00 (4.08)	2.65 (1.75)	0.002^**^

^a^Values shown as mean (standard deviation)

^*^ *p* value was calculated from Student *t*-test

^**^ *p* value from Log BLL

Frequency of BLLs ≥ 5 μg/dL is depicted in Table 4. The proportion of women with BLLs ≥ 5 μg/dL in the exposed group was significantly higher compared to the control group (22.6% vs 7.1%; *p* < 0.01). In addition, women in the exposed group had 3.8 times more probability to have BLLs above 5 μg/dL than those in the control group.

**Table 4:** Frequencies of BLL ≥ 5 μg/dL in the study population

Subjects	BLLs ≥ 5 μg/dL n (%)	BLLs <5 μg/dL n (%)
Exposed group (*n* = 31)	7 (22.6)	24 (77.4)
Control group (*n* = 268)	19 (7.1)	249 (92.9)
Total (*n* = 299)	26 (8.7)	273 (91.3)

*X* ^2^ = 6.56; *p* = 0.010; OR = 3.822; 95%; IC (1.460 – 10.008)

Non-occupational sources of lead exposure for exposed and control groups are summarized in Table 5. The proportion of women who had the habit of dyeing their hair was significantly higher in exposed women when compared to the control group (*p* = 0.010) and the same was observed in the exposed group regarding living near workplaces where lead is used when compared with control women (*p* = 0.043). However, there were no significant differences in other variables between the compared groups.

**Table 5:** Comparison of non-occupational sources of lead exposure between exposed and control groups^a^

Potential source of lead exposure	Exposed group (*n* = 31)	Control group (*n* = 268)	*p* value^*^
Washing the workwear together with other clothes	12 (38.7)	123 (45.9)	0.447
Use of lead glazed pottery	10 (32.3)	81 (30.2)	0.816
Dyeing hair	27 (87.1)	172 (64.2)	0.010
Living near workplaces where lead is used	22 (71.0)	139 (51.9)	0.043
Living near mining zone	4 (12.9)	25 (9.3)	0.752
Living near battery workshop	7 (22.6)	43 (16.0)	0.356
Living near junkyard	4 (12.9)	30 (11.2)	0.777
Living near rubbish dump	3 (9.7)	39 (14.6)	0.641
Living near straightening and painting workshop	7 (22.6)	45 (16.8)	0.421
Pica behavior	10 (32.3)	62 (23.1)	0.261
Living with someone who works with lead	16 (51.6)	101 (37.7)	0.133

^a^Values shown as frequency (percentage)

^*^ *p* value from Chi-square test

To evaluate the influence of some work conditions on blood lead levels in the exposed group, some protection habits were explored (Table 6). Blood lead levels were significantly higher in women who did not wear special workwear (*p* = 0.028) and in those who did not have the habit of changing clothes after work (*p* = 0.025).

**Table 6:** Comparison of blood lead levels regarding protection habits in exposed women

Protection habits	Blood lead levels, μg/dL ^a^		*p* value^*^
	No	Yes	
Use of respiratory protective equipment	27 (4.03 ± 4.23)	4 (3.75 ± 3.43)	0.901
Wearing gloves habit	19 (4.32 ± 4.96)	12 (3.48 ± 2.18)	0.521
Wearing of special workwear	20 (4.92 ± 4.85)	11 (2.31 ± 0.68)	0.028
Hand washing before eating	11 (3.55 ± 1.48)	20 (4.24 ± 5.00)	0.571
Changing clothes after work	24 (4.51 ± 4.52)	7 (2.24 ± 0.60)	0.025
Use of any protective equipment	9 (5.64 ± 7.03)	22 (3.32 ± 1.83)	0.356

^a^ Values shown as frequency (mean ± standard deviation)

^*^ *p* value from Student *t*-test

Table 7 displays potential sources of blood lead in the exposed group. After multivariable analysis, seven variables were retained in the final model: wearing of special workwear, changing clothes after work, living near a painting store, living near a printing office, living near a junkyard, living near a rubbish dump and washing the workwear together with other clothes. These variables accounted for 86.5% of the total variance. The model was adjusted by age, educational level and gestational age.

**Table 7:** Regression analysis for predictors of BLLs in exposed group (*N* = 31)

Variable	Coefficient β	95% CI	P*
Wearing of special workwear	- 0.608	- 1.115 – -0.102	0.021
Changing clothes after work	- 0.637	- 1.261 – - 0.013	0.046
Living near painting store	3.937	1.174 – 6.699	0.008
Living near printing office	7.418	.963 – 10.873	0.001
Living near junkyard	3.661	0.691 – 6.632	0.019
Living near rubbish dump	3.469	0.036 – 6.901	0.048
Washing the workwear together with other clothes	2.372	0.267 – 4.477	0.029

*R* ^*2*^ = 0.865

* Adjusted by age, educational level and gestational age

## Discussion

In this cross-sectional study, we examined the association of blood lead levels with occupational exposure in pregnant women. The blood lead levels in our study population (2.79 ± 2.14 μg/dL) did not exceed the accepted threshold of 10 μg/dL. They are even below the 5 μg/dL recommended by the CDC [31]. Furthermore, the mean blood lead level in our test subjects is lower compared to values reported in some populations of pregnant women. A study by Taylor et al. [14] reported mean BLL of 3.67 ± 1.47 μg/dL in a cohort of pregnant women in The United Kingdom. In China, the lead concentrations during the three pregnancy trimesters and postpartum were 5.95 ± 2.27 μg/dL, 5.51 ± 1.93 μg/dL, 5.57 ± 1.85 μg/dL, and 6.88 ± 1.90 μg/dl; respectively [32]. In addition, Gerhardsson and Lundh [33] reported median blood lead of 11.0 μg/L (range 4.2–79 μg/L) in pregnant females residing in Sweden; and Alvarez et al. [34] found a blood lead average of 11.63 ± 4.64 μg/dL in pregnant women living in the island of Tenerife, Spain. However, some researchers have reported lower blood lead concentrations in pregnant women. Mean blood lead levels of 2.551 ± 2.592 μg/dL were found in pregnant women from Saudi Arabia [35]. In a socioeconomically disadvantaged population of New York, a geometric mean of 1.58 μg/dL was reported by Schell et al. [15]. Moreover, Bakhireva et al. [36] found mean blood lead of 1.06 ± 1.55 μg/dL in a cross-sectional study designed to ascertain risk factors of lead exposure among pregnant women in New Mexico, United States.

In Mexico, the Secretary of Health is the health care institution which attends the smallest workforces. Nevertheless, we found 31 women working in places where lead is used and who represent 10.4% of the recruited subjects. In spite of this, lead in the workplace results a significant determinant of blood lead levels. Therefore, similar results may be expected in other pregnant populations with low income and low level of employment.

Our exposed group was made up of women who worked in automotive repair shops, mining laboratories, welding workshops, automotive harness factories, hairdressing salons, and as road sweepers, regardless of intensity and exposure time. At any rate, we found significantly higher blood lead concentrations in exposed women than in the control group (4.24 ± 4.60 μg/dL vs. 2.66 ± 1.73 μg/dL). Our findings are consistent with a study by Popovic et al. [37], who found mean blood lead of 2.73 ± 2.39 μg/dL in women formerly working in a smelter, and 1.25 ± 2.10 μg/dL in women with no known occupational exposure to lead.

In the present study, no difference was observed in hemoglobin level between exposed women and the control group. This is expected considering the low BLLs obtained for this population. According to previous studies, lead anemia appears at BLLs higher than 40 μg/dL [3, 38]. On the other hand, the US Environmental Protection Agency (EPA) suggests a threshold BLL of 20 – 40 μg/dL for risk of anemia [39]. However, blood lead concentrations in our compared groups are much lower.

Recent findings concerning lead-related adverse reproductive outcomes suggested that pregnant women should avoid lead exposure that would result in blood lead concentrations higher than 5 μg/dL [3]. Among the 299 women included in our study, 26 (8.7%) had BLLs ≥ 5 μg/dL. In a cohort of 4, 285 pregnant women, Taylor et al. [14] reported 14.4% of women with BLLs of 5 μg/dL or higher; cigarette smoking, alcohol, and coffee drinking were found to be predictors of BLLs. However, in our study the frequencies of smoking, alcohol and coffee drinking among the women were very low; therefore, these variables were not included in the analysis. Regarding occupation, the 2005 – 2007 Adult Lead Epidemiology and Surveillance (ALES) by the United States of America Centers for Disease Control and Prevention reported that 32% of women of childbearing age with BLL ≥ 5 μg/dL were occupationally exposed to lead [38]. Zhu et al. [40] evaluated reasons for testing and potential sources of exposure among women, and reported that 29.2% of women with blood lead of 5–14.9 μg/dL had a job with potential lead exposure.

Our results indicated that exposed women were more than 3.8 times likely to have BLLs ≥ 5 μg/dL than non-exposed women. This finding suggests that occupation represents an important factor for elevated blood lead concentrations in our studied population. According to a study by Kosnett et al. [3], it is recommendable for pregnant women to avoid lead exposure that would result in blood lead levels above 5 μg/dL, due to the raised concerns regarding the toxicity of this blood lead concentration. Several studies have associated blood lead levels above 5 μg/dL with miscarriage [19, 20], pregnancy hypertension [12, 21–24, 41], premature delivery [13], premature rupture of the membranes [25], and low birth weight [26, 27]. According to CDC recommendations [28], pregnant women with a current or past BLL ≥5 μg/dL should be assessed for the adequacy of their diet and provided with prenatal vitamins, calcium and iron supplements.

We found a higher proportion of women living near workplaces where lead is used among exposed women compared with the control group. There was also a significant association between the BLLs and the habit of dyeing the hair. Some hair dyes may contain lead and other harmful substances. Our results agree with Marzulli [42] who reported a significant correlation between blood lead and hair lead in people who used lead contained hair dyes. Use of these products by a pregnant woman may harm the health of her unborn child. None of the cited investigations, carried out in an occupational cohort, analyzed non-occupational exposure. However, our findings suggest that the contribution of non-occupational activities must be explored for determining total lead exposure and subsequent health effects.

Occupational lead exposure can occur because of the use of lead material and products. For that reason, employers should provide their employees with adequate working conditions and protection information regarding hazards at their worksites. Exposed workers should use protective equipment and practice personal hygiene, such as showering and changing into clean clothes at the end of the shift [43]. In this study, working women who did not change their clothes after work showed significantly higher blood lead concentration in comparison with those women who had this habit. There was also statistical association of BLLs related to the use of special workwear. It is well known that appropriate workwear can greatly reduce exposure to hazardous substances [44]. In addition, clothing contaminated with lead can be an important route of exposure for pregnant women.

Despite the scientific data and practical considerations regarding the prevention of lead exposure during pregnancy, routine blood lead testing for pregnant women is not established in many countries. Nevertheless, it is the main way to make sure that women have not been affected by lead. Furthermore, some researchers have demonstrated that lead exposure during pregnancy affects children’s physical neonatal development, and available evidence suggests there are no BLLs without risk of health effects [41].

Relatively little is known about the current prevalence, risk factors, and sources of lead poisoning among pregnant women [45]. Our study identified some risk factors associated with blood lead in occupationally exposed women. Despite the growing evidence that relatively low levels of environmental lead exposure may be associated with adverse pregnancy outcomes, there is no specific regulation in existence regarding occupational lead exposure during pregnancy in Mexico. Therefore, it is necessary to improve engineering controls and personal hygiene to reduce the risk of lead exposure during pregnancy. Much work needs to be done to reduce environmental lead exposure. Furthermore, exposed women should undergo blood lead testing to prevent lead poisoning.

We have recognized that our study has several limitations. First, the cross-sectional design did not allow an evaluation of the length and the extent of the exposure. Consequently, all the exposed women were included in a single group, regardless of the time spent in the working place. Longitudinal studies are needed to evaluate the changes in blood lead levels during the exposure time. Second, in our study calcium supplementation, dietary iron intake and indicators of iron status were not measured. It has been documented that low calcium intake may contribute to lead mobilization from the maternal skeleton during pregnancy [46] and that calcium supplementation reduces bone resorption [47] and minimizes release of lead from bone stores with subsequent fetal lead exposure [48, 49]. On the other hand, an inverse relationship between body stores of iron and lead retention has also been observed [50, 51]. Nevertheless, to our knowledge, it is the first study on this topic conducted in occupationally exposed pregnant women in Mexico. Therefore, the results of the present research can be used for comparison with future investigations regarding occupational exposure to lead during pregnancy.

## Conclusions

Our results constitute evidence that pregnant women who work in some places where lead products are used may be at risk for presenting higher blood lead levels if they do not use protective equipment and do not practice adequate personal hygiene. The risk increases if women live near some places that are considered sources of lead exposure such as a painting store, a printing office, a junkyard, or a rubbish dump. Additional studies using larger sample sizes and multiple prospective measurements are needed to verify our findings.

## Additional file

Additional file 1: Database: Blood lead levels in pregnant women from Durango, Mexico. (XLS 733 kb)

## Abbreviations

ALES: Adult lead epidemiology surveillance
BLLs: Blood lead levels
BMI: Body mass index
CDC: Centers for disease control and prevention
CI: Confidence interval
EDTA: Ethylenediaminetetraacetic acid
NIST: National Institute for Standard Technology
OR: Odds ratio
SD: Standard deviation
